# A Radioactive-Free Method for the Thorough Analysis of the Kinetics of Cell Cytotoxicity

**DOI:** 10.3390/jimaging7110222

**Published:** 2021-10-23

**Authors:** Claudia Coronnello, Rosalia Busà, Luca Cicero, Albert Comelli, Ester Badami

**Affiliations:** 1 Fondazione Ri.MED, 90133 Palermo, Italy; acomelli@fondazionerimed.com; 2 Department of Research, I.R.C.C.S.-ISMETT, 90127 Palermo, Italy; rbusa@ismett.edu; 3 Istituto Zooprofilattico Sicilia, IZS, 90127 Palermo, Italy; cicero.luca@libero.it

**Keywords:** cytotoxic assay, chromium release assay, fluorescence, bioluminescence imaging, kinetics

## Abstract

The cytotoxic activity of T cells and Natural Killer cells is usually measured with the chromium release assay (CRA), which involves the use of 51Chromium (^51^Cr), a radioactive substance dangerous to the operator and expensive to handle and dismiss. The accuracy of the measurements depends on how well the target cells incorporate ^51^Cr during labelling which, in turn, depends on cellular division. Due to bystander metabolism, the target cells spontaneously release ^51^Cr, producing a high background noise. Alternative radioactive-free methods have been developed. Here, we compare a bioluminescence (BLI)-based and a carboxyfluorescein succinimidyl ester (CFSE)-based cytotoxicity assay to the standard radioactive CRA. In the first assay, the target cells stably express the enzyme luciferase, and vitality is measured by photon emission upon the addition of the substrate d-luciferin. In the second one, the target cells are labelled with CFSE, and the signal is detected by Flow Cytometry. We used these two protocols to measure cytotoxicity induced by treatment with NK cells. The cytotoxicity of NK cells was determined by adding increasing doses of human NK cells. The results obtained with the BLI method were consistent with those obtained with the CRA- or CFSE-based assays 4 hours after adding the NK cells. Most importantly, with the BLI assay, the kinetic of NK cells’ killing was thoroughly traced with multiple time point measurements, in contrast with the single time point measurement the other two methods allow, which unveiled additional information on NK cell killing pathways.

## 1. Introduction

Cell-mediated killing is a pivotal mechanism in host immune responses to pathogens. Natural Killer (NK) cells and cytotoxic T lymphocytes (CTLs) represent the two main types of cytotoxic cells. NK cells are the main players of innate immunity and interact with target cells via inhibitory and activating receptors. CTLs are restricted by the major histocompatibility complex (MHC) molecules and utilize the T cell receptor to recognize antigenic peptides. Both cytotoxic cells kill the target by inducing cell lysis through the release of perforin and granzyme A/B and through the Fas–Fas ligand pathway [1].

For in vivo proof-of-concept and clinical evaluation of the efficacy of cell-mediated immunotherapy, it is mandatory to assess the effector functions of NK cells and CTLs against tumor target cells. In addition, a more accurate understanding of the kinetics of cell-to-cell killing might help to design more appropriate experimental protocols with well-timed data acquisition points. 

The classic chromium release cytotoxicity assay is extensively used to measure the cytotoxic activity of effector cells in vitro and still represents the gold standard in measuring CTL- or NK cell-mediated cytotoxicity [2]. However, the chromium release assay has several negative aspects to consider, as ^51^Cr is radioactive and therefore harmful to the operator’s health. ^51^Cr handling requires special radioactive training, facilities, and safe disposal, which comes at elevated costs. In addition, due to spontaneous death or spontaneous release, ^51^Cr assays might suffer considerable background noise, which also depends upon the nature of the target cells, as some cell types produce higher background than others. Thus, safer and alternative methods to measure the cytotoxicity of cells are needed.

Some alternative methods have been developed in order to exclude the use of radioactive substances. For instance, flow cytometric assays use fluorescent dyes such as carboxyfluorescein succinimidyl ester (CFSE) [3], bromodeoxyuridine (BrdU) [4], the lipophilic membrane dye PKH26 [5], or the amine-reactive dyes Live/Dead of commercial assays [6]. These dyes can be combined with other antibodies for multiple color analysis via flow cytometry to measure cytotoxicity and can reproduce with high fidelity the data obtained with the chromium assay. However, these dyes might be toxic to the cells and alter their survival, as observed for CFSE [7]. The BrdU staining protocol is lengthy and might induce DNA damage [8]; results of the Live/Dead assay might be characterized by a high background due to natural cellular autofluorescence and the irreversibility of the reaction [6].

In this study, we used a different approach exploiting the bioluminescence (BL) of a tumor cell line stably expressing the firefly luciferase enzyme. BL imaging (BLI) is a technique based on the detection of photons emitted by cells that express the enzyme luciferase, commonly used as a reporter gene. The firefly luciferase catalyzes the reaction of d-luciferin with O^2^ to produce light in the presence of Mg^2+^ and ATP, as occurs in cells with active metabolism [9]. Luciferase is not expressed by mammalian cells unless genetically modified. As a consequence, background noise is very low, as photons are emitted only by cells specifically expressing the reporter gene.

Instruments such as luminometers or live imaging spectrometers detect photons emitted by luciferase-positive target cells when the substrate d-luciferin is provided in the culture medium. d-luciferin oxidation by luciferase emits a signal that is directly proportional to the number of cells that express the enzyme [10]. Thus, in a killing assay, NK and CTL cellular cytotoxicity can be detected as a decrease in the emission of photons by the target cells [11].

Besides the above-mentioned issues related to radioactivity, i.e., cell toxicity and high background noise, assays based on ^51^Cr incorporation and immunofluorescence are usually limited to a single time point readout, thus rendering these methods non-applicable for the execution of fine kinetics studies. A thorough dissection of the kinetic of cell killing might provide information that in a single-time point assay would remain undiscovered. 

In this study, we provide evidence of the advantages of using BLI compared to the classic ^51^Cr release assay or fluorescence-based killing assays using an in vitro model of human hepatocellular carcinoma stably expressing the firefly luciferase and cytotoxic NK cells. Here, we show that the use of the IVIS Spectrum imaging system allows the acquisition of multiple serial photon emission signals which unexpectedly unveil additional information.

## 2. Materials and Methods

### 2.1. Ethic Statement

Lymphocytes were isolated from the backtable fraction of the product of perfusion of the liver excised from brain-dead–heart-beating donors. The study was conducted in accordance with the principles outlined in the Declaration of Helsinki of 1996, and ISMETT’s Institutional Research Review Board approved the protocol (protocol number IRRB/14/15, 7 April 2015).

### 2.2. Cell Lines, Primary Cells, and Culture Conditions

The HepG2 human hepatocellular carcinoma cell line was purchased from the Lombardy and Emilia Romagna Experimental Zootechnic Institute (BS TCL 79, Brescia, Italy). The HepG2-Red-FLuc Bioware^®^ Brite Cell Line (BW134280, Perkin Elmer, Waltham, MA, USA) is a light-producing cell line derived from HepG2. The cells have been stably transduced with the red-shifted firefly luciferase gene and expanded with EMEM (ATCC), containing 10% heat-inactivated fetal bovine serum (FBS; HyClone, Logan, UT, USA). Under a laminar hood, liver graft perfusates were transferred from the ATR into 500 mL conical tubes (Corning, GmbH HQ, Wiesbaden, Germany) and centrifuged at 2000 rpm at 10 °C, for 10 min in the Heraeus Multifuge 4KR DJB (Labcare Ltd, Newport Pagnell, UK). Contaminating erythrocytes were lysed using red lysis buffer ACK (home-made) for 5 min at room temperature. Cells were washed twice with Dulbecco’s D8537 phosphate-buffered saline (Sigma-Aldrich s.r.l, Milan, Italy) containing 2% fetal bovine serum (Hyclone), and cell number and vitality were obtained by 17-942E trypan blue exclusion (Lonza, Basel, Switzerland). Primary CD3-CD56+ NK cells were isolated from the backtable fraction of liver perfusate using a human NK cell isolation kit (Miltenyi Biotec, Bergisch Gladbach, Germany) following the manufacturer’s instructions. In all experiments, only fractions with a purity above 95% were used. Two million cells were seeded in 12-well plates and maintained in culture with NK cells Activation/Expansion Beads following the manufacturer’s instructions (Miltenyi Biotec) in NK cell MACS medium (Miltenyi Biotec) containing IL2 at 500 IU/mL (Proleukin) and IL15 at 20 ng/mL (Miltenyi Biotec).

### 2.3. Chromium Release Assay

The chromium (^51^Cr) release assay was used to measure the cytotoxicity of NK cells. HepG2 were used as target cells. We plated 150,000 target cells in a 6-well plate in 1 mL of medium supplemented with 50% FBS, and 100  μCi ^51^Cr was added. Cells were incubated at 37 °C overnight. The target cells were washed twice with RPMI 1640 (Sigma-Aldrich) supplemented with 10% heat-inactivated FBS (R-10), resuspended in 10 mL of R-10, and left to rest at room temperature for 30 min to release the last residual chromium. Cells were plated in 0.1 mL R-10 at a concentration of 1000 cells per well in a 96-well round-bottom plate. As effectors, NK cells were resuspended in R-10 and added to the wells in appropriate dilutions to obtain the predetermined effector-to-target (E/T) ratios of 40:1, 20:1,10:1, 5:1, 2.5:1, and 1.25:1 in a final volume of 0.1 mL. Negative-control wells contained the target cells alone for measuring spontaneous ^51^Cr release, and positive-control wells contained the target cells incubated with 0.1 mL 10% Triton X-100 (Sigma-Aldrich) for measuring the maximum ^51^Cr release. Cells were seeded in triplicate. After a 4 h incubation, ^51^Cr release was measured with a McroBeta2 counter (PerkinElmer), and the percent of target cell lysis was calculated as follows: (mean experimental counts per minute (cpm) − mean spontaneous cpm)/(mean maximum cpm − mean spontaneous cpm)  ×  100%.

### 2.4. Flow Cytometry-Based Killing Assay

HepG2 target cells were labelled using the CellTrace™ CFSE Cell Proliferation Kit for flow cytometry, following the manufacturer’s instructions (Molecular Probes). CFSE-labelled HepG2 target cells were plated at 40:1, 20:1,10:1, 5:1, 2.5:1, and 1.25:1 or at a 1:0 ratio with effector NK cells for 4 h, and target cell viability was detected by fluorescence with the 7AAD dye (Miltenyi Biotec, dilution 1:10). NK cells were counterstained with APC-CD56 (IgG1, clone TULY56, from eBioscience, San Diego, CA, USA, dilution 1:10).

### 2.5. BLI-Based Cytotoxicity Assay

Luciferase-expressing HepG2-Red-FLuc tumor cells were plated in black 96-well flat-bottom microplates (Corning). After the addition of the IVISbrite d-luciferin Potassium Salt Bioluminescent Substrate (XenoLIght™ d-luciferin K+ salt) (150 ug/mL; Perkin Elmer), photon emission was measured with the IVIS Spectrum In Vivo Imaging System (Perkin Elmer).

To set up the experimental conditions, we first determined the minimal number of cells per well required to obtain a signal of at least one log above the background. We plated in triplicate a decreasing number of HepG2-Red-FLuc cells (2.50 × 10^5^ to 1 × 10^2^) in black 96-well plates (Corning). After the addition of d-luciferin (final volume per well 0.350 mL), emission was determined. The signal was above the background and linear with the number of cells in the whole analyzed range (adjusted R-squared = 0.998). We then established that a minimum number of target cells to detect a stable signal was 1 × 10^4^ cells/well (data not shown).

To perform the experiments, we plated 5 × 10^4^ target cells/well, and effector NK cells were added at 40:1, 20:1,10:1, 5:1, 2.5:1, and 1.25:1 effector-to-target (E:T) ratios; the cells were incubated at 37 °C in the IVIS reading screen. After 10 min from d-luciferin addition, cytotoxicity was traced live by acquiring images every 2 min for the first hour and every 5 min for the following 3 h.

A 12 × 8 grid of squared regions of interest (ROI) was placed over the plate image, in order to contain each well into a squared ROI. The total signal (photons/s) in each ROI was determined by the Living Image software. The same grid of ROIs was applied in each acquisition.

### 2.6. Data Analysis

In each of the three assays, the cytotoxic effects 4 hours after NK cell addition were plotted at different NK/target cells ratios. In addition, in the BLI assay, the total signal intensity vs. time after d-luciferin addition was plotted to obtain the time–intensity curve for several NK/target cells ratios. In each time–intensity curve, the peak intensity was obtained as the maximum reached signal. Peak intensity and the intensity 4 hours after NK addition were plotted against the NK:target cells ratios. The intersection time T_int_ was obtained as the time at which the time–intensity curve intersects the control curve, and the obtained values were plotted against the NK:target cells ratios.

## 3. Results

### 3.1. NK Cells Cytotoxicity Measurement by the Chromium Release Assay

The killing function of cytotoxic NK cells was determined by cytotoxicity assays using tumor target cells labelled with radioactive ^51^Cr, co-cultured at increasing ratios of NK effector cells at an effector-to-target (E:T) ratios ranging from 40:1 to 1:1 over a period of 4 h. The percentage of cytotoxicity was calculated by measuring the target cells’ viability, which in classic chromium release assays, is determined by the amount of radioactive chromium released in the supernatant.

In this study, primary human NK cells, derived from five healthy donors, were challenged with the human hepatoma-derived cell line HepG2. NK cells were expanded and maintained in vitro with cytokines IL2 and IL15 until performing the assay, while the target cells were labelled overnight with radioactive ^51^Cr. After 4 h of co-incubation, the killing activity was therefore measured by taking into account the amount of ^51^Cr spontaneously released by the target cells, expressed by the formula

$$\mathrm{Specific}\;\mathrm{lysis}\;\left(\%\right)=\frac{\bar{\mathrm{experimental}\;\mathrm{cpm}}-\bar{\mathrm{spontaneous}\;\mathrm{cpm}}}{\bar{\mathrm{maximum}\;\mathrm{cpm}}-\bar{\mathrm{spontaneous}\;\mathrm{cpm}}}\times 100$$

where cpm = counts per minute.

**Figure 1:**
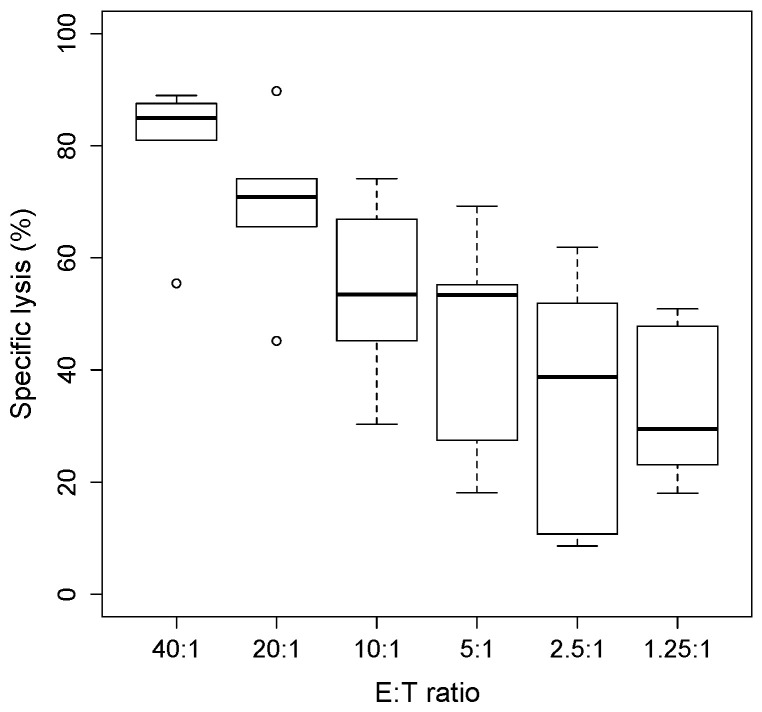
Chromium Release Assay. Chromium release assay (4 h) of IL2/IL15-NK challenged with HepG2 target cells labelled overnight with 5μCi of radioactive 51Cr. The percentage of specific lysis was determined by measuring the amount of chromium released by dying cells in the supernatant, using the formula: lysis % = (mean experimental counts per minute (cpm) − mean spontaneous cpm)/(mean maximum cpm − mean spontaneous cpm)  ×  100%. Each boxplot describes the % lysis obtained with cells from five donors.

As it is shown in Figure 1, we found that increasing the NK/target cells ratio increased the cytotoxicity against HepG2 target cells, expressed as % of cell lysis.

### 3.2. NK Cells Cytotoxicity Measurement by Flow Cytometry

**Figure 2:**
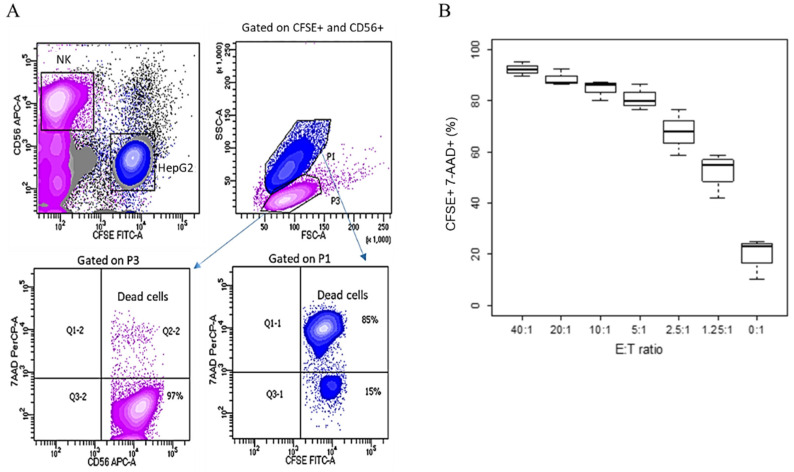
Fluorescence-based cytotoxicity assay. HepG2 target cells were labelled with CFSE 5 µM and co-cultured with decreasing ratios, from 40:1 to 0:1, with effector IL2/IL15-NK cells for 4 h; cell killing was determined by flow cytometry. (**A**) Gating strategy. NK cells were marked as CD56+, target HepG2 cells were CFSE+. On an FSC/SSC plot gated on CD56+ and CFSE+, the two subpopulations were morphologically distinct. HepG2 (gated on P1) expressed 7AAD. By contrast, NK cells (gated on P3) were 7AAD-negative. Contour plots show one representative experiment out of three, with NK cells added to HepG2 at a 2.5:1 ratio. (**B**) Cumulative plot. At 4 h, the % of CFSE+7AAD+ dead target cells was determined by flow cytometry. The experiment was repeated three times using NK cells from different donors, and the % of dead target cells was plotted in floating boxes, where the median is indicated.

An alternative method to investigate cells mortality in a killing assay is based on the use of fluorescent dyes. In this case, HepG2 target cells were labelled with CFSE and, similarly to the previous experiment, NK cells were expanded with IL2/IL15 until use. Effector NK cells were added at decreasing E/T ratios from 40:1 to 0:1, and after 4 h, the cell mix was stained with a CD56 antibody, recognizing a specific marker for NK cells [12]. To assess cell mortality, the cells were counterstained with 7-amino-actinomycin D (7-AAD), discriminating viable from apoptotic cells [13]. Figure 2A shows the gating strategy that we used. NK cells were identified as CD56+. The target cells were those that stained positively for CFSE. In an SSC/FSC plot, used to analyze cell morphology, the two populations appeared neatly separated, with HepG2 target cells larger than NK cells, as expected. The percentage of dead cells in each population was determined by gating either on CFSE+ or on CD56+.

When IL2/IL15 NK cells were added to the culture, a population of CFSE+ target cells was clearly distinguishable after 4 h of co-culture, but the percentage of cells that also stained positively for 7AAD increased with the number of killer cells added (Figure 2B). We also observed that NK cells were virtually 100% vital, as they were negative for 7AAD (Figure 2A).

Due to its lipophilic characteristics, CFSE integrates into the cell membrane and enters the cytoplasm, conferring fluorescence to the cells. CFSE staining is cheap and easy to perform; however, it can also be toxic to the cells. As the endpoint of our investigation was comparing alternative and radioactive-free protocols to the ^51^Cr assay for the determination of NK cells’ killing activity, a possible toxic effect on the target cells due to CFSE staining might have produced artifacts and made target cells more susceptible to cell death prior to NK cells addition, possibly increasing the background noise. This was also demonstrated by the presence of 7AAD+ target cells in the wells where NK cells were not added.

### 3.3. NK Cells Killing Detection by Bioluminescence Imaging

Lastly, we tested an alternative and, to our knowledge, unexplored approach to performing a cytotoxic assay based on Bioluminescence Live Imaging measured with the In Vivo Imaging System IVIS Spectrum (Perkin Elmer). Importantly, both ^51^Cr- and fluorescence-based assays are usually carried out at a single time point, while the use of bioluminescence provides the automatic acquisition of multiple time points measures over a long period of time, providing a more accurate understanding of the cytotoxic function of NK cells and CTLs.

**Figure 3:**
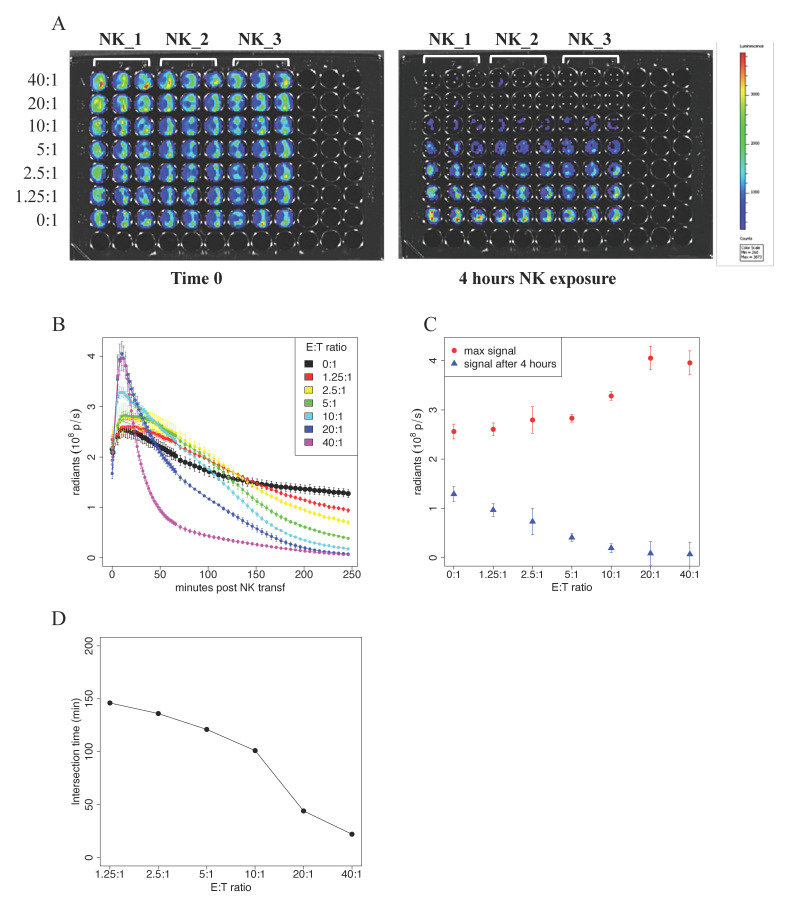
BLI-based cytotoxicity assay. In vitro quantitation of the bioluminescence signal in human hepatocellular carcinoma cells expressing luciferase, HepG2-Red-FLuc. (**A**) Pseudocolor representation at time 0 and after 4 h of the bioluminescence intensity of HepG2-Red-FLuc plated at 5 × 10^4^/well in a cytotoxicity assay where human NK cells from three different patients (NK_1, NK_2, and NK_3), activated with IL2/IL15, were added at decreasing ratios ranging from 40:1 to 1.25:1. (**B**) the thorough kinetic of cytotoxicity was addressed by measuring photon emission every two minutes for the first hour and subsequently every 5 min up to 4 h of total incubation. Average signal values and standard errors were plotted against time post NK cell incubation. (**C**) Peak signal and signal 4 h after NK injection plotted against NK concentration. (**D**) Intersection time, i.e., the time at which the time–intensity curve intersects the control time–intensity curve, plotted against NK concentration. (**A**–**D**) Data shown are representative of three experiments performed in similar conditions using NK cells isolated from three different donors.

Here, the target cells were represented by the hepatocellular carcinoma cell line stably expressing the enzyme Luciferase, i.e., HepG2-Red-FLuc. In this assay, cell vitality is measured by the acquisition of photon emission upon the addition of the substrate of the Luciferase enzyme, d-luciferin. In our experiments, 5 × 10^4^ HepG2-Red-FLuc cells were seeded in black 96-well plates, and decreasing ratios (from 40:1 to 1.25:1) of IL2/IL15-activated NK cells were added. After the addition of d-luciferin, photon emission was measured every 2 min during the first hour, and then every 5 min for the following 3 h. In Figure 3A, we report pseudocolor snapshots of the plates acquired at time 0, where target cells are alive, and a homogenous signal is clearly visible. After 4 h from the addition of NK cells, a loss of photon emission in the wells where NK cells were added at the highest ratios was appreciable.

The plot of photon emission signal vs. time post NK addition is reported in Figure 3B. The figure shows a transient increase of the signal in the first 10 min in all the samples. The reached peak is higher the higher is the concentration of NK cells, with few exceptions, as reported in Figure 3C. After the first 10 min, signal emission decreased and reached the same level as that of the control sample at a certain Time of Intersection (T_int_); this occurred faster for higher NK cells concentrations. The value of T_int_ obtained at different NK cells concentrations is plotted in Figure 3D. The signal value at 240 min after NK addition is reported in Figure 3C. These are the only data point that can be compared with the results obtained with the previous experimental approaches. The data were consistent with those previously obtained with the Chromium Release Assay and Flow Cytometry technique.

## 4. Discussion

We applied three experimental techniques to investigate the ability of NK cells to kill cancer cells. Specifically, we compared the results obtained with a chromium release assay, a Flow Cytometry technique, and a bioluminescence assay. With all three techniques, we were able to assess NK cell killing efficiency at a single time point, specifically, 4 h after NK addition. Consistently, we observed that the percentage of killed cells increased the higher was the relative concentration of added NK cells.

In order to perform a chromium release assay or a Flow Cytometry analysis, the analyzed sample has to be manipulated in an irreversible way, and each sample can be used for a single time point measure only. For this reason, the extension from a single time point measure to a time course requires an increase of the costs proportional to the number of time points to be investigated.

The advantages of using bioluminescence imaging in cytotoxic assays have been described [11]. However, these studies were limited to measures on a single time point and, to our knowledge, a proper time course investigation was left unexplored. The bioluminescence assay, indeed, requires minimal sample manipulation without the addition of cytotoxic reagents. Consequently, BLI allows following photon emission without damaging the sample itself and, therefore, the acquisition of multiple measurements. The obtained results allowed us to speculate about the kinetics of NK cytotoxicity effects on the vitality of the cells from the first minutes after NK addition. Indeed, the IVIS Spectrum In Vivo Imaging System permits to automatically acquire the time course of the bioluminescence signal by choosing the total number of acquisitions and the delay between two consecutive acquisitions.

The bioluminescent photon emission signal 4 h after NK addition was lower the higher was the concentration of NK cells, as also demonstrated by others on a single-time course BLI killing assay [11]. This is due to the killing activity of NK cells over the target cells with consequent reduction of the signal emitted by a lower number of living cells. Surprisingly, the signal time course in the first minutes after NK addition revealed higher emission the higher was NK concentration. Indeed, the signal of the control sample, i.e., target cells with no addition of NK cells, shows an increase in the first 10 min, followed by a slower decrease. A very similar behavior is reported in in vivo bioluminescence time–intensity curves, with the difference that the maximum signal was reached after more than 10 min [14]. In this case, the increasing signal was due to the rate of absorption of d-luciferin into the blood and its delivery to the tumor cells. In our case, the increase was probably due to the saturation of the intake of the d-luciferin by the target cells, and the decrease was probably due to d-luciferin degradation. We suppose that the introduction of NK cells interferes with the intake of d-luciferin by the cells by increasing their permeability [15].

NK cells’ function is to eliminate virally infected and tumorigenic cells. To do this, they store cytotoxic proteins within secretory lysosomes, also known as lytic granules. Upon recognition of an aberrant target cell, polarized exocytosis of secretory lysosomes is activated with the release of cytotoxic molecules and the killing of the target cell [16]. Secretory lysosomes have a degradative function and contain major cytotoxic proteins such as granzyme A/B and perforin. Target cell recognition induces secretory lysosome exocytosis and the release of their cytotoxic content. Perforin then facilitates the entry of granzymes into the target cell’s cytoplasm, where they cleave a variety of targets, such as caspases, resulting in the death of the target cell [16]. We postulate that upon addition to the assay, NK cells recognize the tumor target cells, with the consequent triggering of the killing pathway mediated by cytotoxic granules. This increases cell permeability and consequently speeds d-luciferin intake by the target cells, thus explaining the augmented photon emission in the first minutes of the killing assay.

To conclude, further experiments are needed to unravel the biological interactions underneath the transient behavior of bioluminescent photon emission after NK addition in in vitro experiments. Nevertheless, we believe that the use of the described bioluminescent assay to in vitro monitor the kinetics of NK cells’ cytotoxic effects will enrich the available tools useful to evaluate NK cells efficiency in killing cancer cells targets and will help to better understand how to translate in vivo these experiments.

## Data Availability

Not applicable.
